# Integration of Solid State and Submerged Fermentations for the Valorization of Organic Municipal Solid Waste

**DOI:** 10.3390/jof7090766

**Published:** 2021-09-16

**Authors:** Gheorghe-Adrian Martău, Peter Unger, Roland Schneider, Joachim Venus, Dan Cristian Vodnar, José Pablo López-Gómez

**Affiliations:** 1 Institute of Life Sciences, University of Agricultural Sciences and Veterinary Medicine, Calea Mănăştur 3-5, 400372 Cluj-Napoca, Romania; adrian.martau@usamvcluj.ro; 2 Faculty of Food Science and Technology, University of Agricultural Sciences and Veterinary Medicine, Calea Mănăştur 3-5, 400372 Cluj-Napoca, Romania; 3 Leibniz Institute for Agricultural Engineering and Bioeconomy, Max-Eyth-Allee 100, 14469 Potsdam, Germany; punger@atb-potsdam.de (P.U.); rschneider@atb-potsdam.de (R.S.); jvenus@atb-potsdam.de (J.V.)

**Keywords:** solid state fermentation, enzyme production, wheat bran, organic fraction of municipal solid waste, hydrolysis, *Aspergillus awamori*, *Bacillus coagulans*, lactic acid

## Abstract

Solid state fermentation (SsF) is recognized as a suitable process for the production of enzymes using organic residues as substrates. However, only a few studies have integrated an evaluation of the feasibility of applying enzymes produced by SsF into subsequent hydrolyses followed by the production of target compounds, e.g., lactic acid (LA), through submerged-liquid fermentations (SmF). In this study, wheat bran (WB) was used as the substrate for the production of enzymes via SsF by *Aspergillus awamori* DSM No. 63272. Following optimization, cellulase and glucoamylase activities were 73.63 ± 5.47 FPU/g_ds_ and 107.10 ± 2.63 U/g_db_ after 7 days and 5 days of fermentation, respectively. Enzymes were then used for the hydrolysis of the organic fraction of municipal solid waste (OFMSW). During hydrolysis, glucose increased considerably with a final value of 19.77 ± 1.56 g/L. Subsequently, hydrolysates were fermented in SmF by *Bacillus coagulans* A166 increasing the LA concentration by 15.59 g/L. The data reported in this study provides an example of how SsF and SmF technologies can be combined for the valorization of WB and OFMSW.

## 1. Introduction

The massive generation of solid waste has made their disposal an important challenge to overcome. Global municipal solid waste (MSW) production is expected to increase up to 70% by 2050 from 2010 million tons (Mt) in 2016 [1]. In addition to MSW, substantial global industrialization has also contributed to an unprecedented increase in the generation of industrial solid waste. In 2017, the rate of industrial waste generation was approximately 18 times higher than MSW [2]. A large portion of industrial wastes and MSW are organic residues, which can be used as substrates in fermentations.

The organic fraction of municipal solid wastes (OFMSW) represents an ample category of bio-waste (with an annual production of approximately 140 Mt just in the European Union (EU)) from households, restaurants, diverse businesses, yards and garden wastes, etc. [3,4]. The need for sustainable technologies to manage such abundant residues as the OFMSW and industrial organic wastes must be a priority. Until recently, OFMSW had been landfilled with adverse effects on the environment and wasting the potential of such residue for biotechnological applications.

Even though the composition of OFMSW may vary, these biowastes are typically rich in carbohydrates, proteins and lipids, making them a good substrate candidate for fermentation processes [5]. Most developed countries have efficient OFMSW and industrial organic waste sorting systems and facilities, which has allowed them to use such residues in for biogas production [6,7]. However, biogas value is low and thus, several works have been presented for the valorization of OFMSW through the production of other chemicals, for example, bio-oils, volatile fatty acids subsequently used for biogas production, or polyhydroxyalkanoates [8,9,10,11], hydrogen [12], ethanol, enzymes, organic acid, biopolymers and bioplastics [13,14,15,16,17]. Nonetheless, the polymeric carbohydrates (mainly starch, cellulose and hemicellulose) present in the OFMSW need to be hydrolyzed in order to release simple sugars for the fermentation, which increases costs.

Enzymatic hydrolysis requires mild process conditions, is highly specific and does not generate undesired side products, making it attractive for biowastes hydrolysis. However, the manufacturing of enzymes is expensive, leading to high costs of commercially available enzyme mixtures, raising the production costs of hydrolysis processes [5,18,19,20]. Solid state fermentation (SsF) is a green process that draws attention to enzyme production because it has a low cost, high yield and optimal use of agro-industrial by-products [21]. In SsF, microorganisms grow on a solid substrate with enough water to support microbial metabolism [21,22]. Using SsF as a production method of enzymes could offer some apparent economic and technical advantages over conventional submerged-liquid fermentation (SmF). These include a high product concentration and simple fermentation tools and low necessities for aeration and agitation during enzyme production [23]. Furthermore, SsF can directly use agricultural wastes, thus helping to prevent the negative environmental impact caused by their accumulation. In this context, numerous reports have shown that wheat bran (WB), the most abundant by-product produced from the wheat processing industry, to be a suitable substrate for the production of hydrolytic enzymes by SsF [20,24,25,26].

In recent years, the interest in cellulase and glucoamylase has increased due to the numerous potential applications for these enzymes. Glucoamylase can hydrolyze stepwise single glucose units from the non-reducing ends of amylose and amylopectin from starch or related polymers [27]. It has been extensively used in starch processing and bioconversion of organic waste [28], and many authors have exploited the abundance and availability of agricultural residues to synthesize amyloglucosidase in fermentations. Glucoamylase has been produced from WB, paddy husk, rice bran, wheat flour, cornflower, coconut seed flour, coconut oil cake, tea waste and other starch-containing wastes. *Aspergillus* spp. has been intensively studied for the possibility of using agricultural waste, such as rice bran, for glucoamylase production via SsF [29].

**Figure 1:**
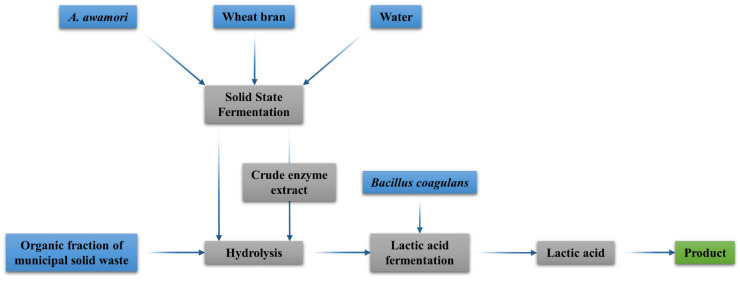
Novel bioprocess for producing enzymes from wheat bran to obtain lactic acid from the organic fraction of municipal solid waste.

Based on the enzyme production from solid organic waste via SsF and their subsequent use in hydrolyses, a novel process has been described [30]. In such process, SsF, hydrolysis and SmF are used consecutively to produce specific compounds and valorize the organic solid wastes, as shown in Figure 1.

The present study aimed to combine SsF, hydrolysis and SmF to valorise the residues WB and OFMSW. This was achieved by (1) using a fungus specialized in enzyme production (*Aspergillus awamori*) via SsF and WB as a substrate (this fungus produces cellulase, glucoamylase and has the highest capacity to produce feruloyl esterase and amylase compared to other *Aspergillus* sp.); (2) the fermented substrate rich in enzymes obtained was used in the hydrolysis of OFMSW to increase sugars; (3) sugars rich hydrolysates were used as the substrate in SmF by a *Bacillus* specialized in L–lactic acid production. Lactic acid (LA) is an important building block with various applications in industry and that has had a renewed interest because its potential application in the polymer and cosmetic market.

## 2. Materials and Methods

### 2.1. Substrates

WB was provided by a farm (SC ALBATROS SRL) in Romania. The OFMSW was kindly delivered from the IMECAL SA company (L’Alcúdia, Valencia, Spain) and was obtained from an MSW treatment plant located in Valencia, Spain. To avoid undesirable fermentation with microorganisms already existing in the substrates, all the substrates were autoclaved for 15 min at 121 °C prior fermentations. The flask’s content for SsF was mixed with a sterile spatula, and in the bioreactor WB and OFMSW mix, homogenization was achieved using the bioreactor mixing system [31]. Before the homogenization of the substrates, OFMSW was screened to remove inert constituents such as stones, plastic, glass, etc. Additionally, sterilized deionized water was used for all experiments. Culture media components for microorganisms’ activation and inoculations and other reagents were of analytical grade, obtained from VWR International (Radnor, Pennsylvania, PA, USA) and agar (Agar plant for cell culture) obtained from AppliChem (Omaha, NE, USA).

### 2.2. Microorganisms and Culture Conditions

The experiments were conducted using *Aspergillus awamori* DSM No. 63272 and WB as a substrate for enzyme production. *Bacillus coagulans* A166 and OFMSW substrate for hydrolysis process and LA fermentation. All microorganisms were obtained from the Leibniz Institute for Agricultural Engineering and Bioeconomy (ATB) in Potsdam, Germany.

The propagation for *A. awamori* occurred in commercial potato dextrose agar (PDA) containing infusion from potatoes, 20 g/L glucose and 15 g/L agar. On the solidified agar, 100 µL pre-inoculum with spore suspension spread evenly with a glass Drigalsky spatula on the agar surface. For inoculation, 0.1% Tween 80 solution and sterile glass beads were used to remove spores from the agar plates. The plates inoculated were incubated at 30 °C for 5 days until the entire plates formed a uniform mass of black spores.

Pre-cultures for LA fermentations were passed in 250 mL shake flasks containing de Man, Rogosa and Sharpe (MRS) medium (glucose, 20.00 g/L; casein peptone, tryptic digest, 10.00 g/L; meat extract, 10.00 g/L; yeast extract, 5.00 g/L; Na-acetate, 5.00 g/L; K_2_HPO_4_, 2.00 g/L; (NH_4_)_3_ citrate, 2.00 g/L; Tween 80, 1.00 g/L; MgSO_4_ × 7 H_2_O, 0.20 g/L; MnSO_4_ × H_2_O, 0.05 g/L) a specific liquid medium suggested for use in the cultivation of *Lactobacillus* spp., and dolomite EVERZIT Dol 0.5–2.5 mm (Evers, Germany) [32]. After inoculation, the flasks were incubated at 150 rpm and 52 °C for 12–16 h [7]. The medium used for *Bacillus coagulans* was MRS broth with a final pH of 6.

### 2.3. Solid State Fermentation, Hydrolyses and Lactic Acid Fermentation

#### 2.3.1. Solid State Fermentation

WB was used for enzyme production via SsF, with an initial moisture content of 11.978 ± 0.05%, was adjusted to 80% using deionized sterile water. A 400 µL solution (10^7^ spores/mL) of *A. awamori* was used to inoculate 10 g of WB in 250 mL shake flasks. The flasks content was mixed with a sterile spatula and incubated at 30 °C for 4 days. A whole flask was taken for each day of fermentation for sample analysis and evaluation of the SsF. The flask content was mixed and homogenized with a spatula, and then the required amount of fermented substrate was taken. Spores’ suspension concentration was measured using a microscope (Zeiss, Oberkochen, Germany) and a Thoma cell counting chamber. Cellulase activity, glucoamylase activity, total reducing sugar test and pH value were monitored. In addition, the samples taken for the total reducing sugar test and pH were inactivated in a water bath for 10 min at 95 °C and kept frozen at −18 °C until further analysis [33].

#### 2.3.2. Hydrolysis Processes

A hydrolysis optimization was made in 300 mL flasks at 50 °C and 150 rpm of shaker without the pH adjusted for 68 h. Five samples were prepared with concentrations of fermented WB rich in enzymes (WBE) and OFMSW at ratios 1:9, 2:8, 3:7, 4:6 and 5:5 (mass/mass). The best ratio obtained previously was used for hydrolysis in a 1.5 L bioreactor (Sartorius Stedim Biotech AG, Göttingen, Germany) with a working mass of 1 kg. The temperature for hydrolysis in the bioreactor was 50 °C with 150 rpm of stirring and a pH 5, controlled by the addition of NaOH (20% *w*:*w*). The results obtained during the hydrolysis with WBE were compared to those obtained using a commercial enzymatic (CE) cocktail (Cellic CTec2; Novozymes A/S, Basgsværd, Denmark), a cellulose complex for the degradation of cellulose into fermentable sugars. The CE mixture was added at 1% (10 mL of CE for one Kg of the substrate). The hydrolysis lasted for 68 h, and samples were taken at times 0, 24, 48 and 68 h to quantify the formation of sugars. In addition, the experiments were made in triplicate, and the samples taken for the total reducing sugar test and pH were inactivated in a water bath for 10 min at 95 °C, after which kept in the freezer at −18 °C until further analysis.

#### 2.3.3. Lactic Acid Fermentation

LA fermentations were carried out successively after hydrolyses in the same 1.5 L BIOSTAT bioreactors. The fermentation parameters were adjusted at 52 °C, 200 rpm for 25 h and a pH of 6.0 was controlled by the addition of NaOH (20% *w*/*w*). Samples were taken every two hours to quantify glucose, fructose, disaccharide, xylose, arabinose, lactic and acetic acids. Cell viability was measured by spread plate method on MRS agar. The experiments were made in duplicate, and the samples taken for the sugars and acids quantification were inactivated in a water bath for 10 min at 95 °C and kept in the freezer at −18 °C until further analysis.

### 2.4. Enzymatic Extract

After appropriate culturing periods, the enzymes produced via SsF were extracted. The crude enzymatic extract was prepared by adding 5 g of the homogenized fermented sample (the whole flask homogenization was carried out with a sterile spatula for 5 min) and 40 mL of deionized water in a 250 mL flask. The flask was then placed on a rotary shaker (150 rpm, 30 min at room temperature). To collect the enzymatic extract, the samples were centrifuged at 4800× *g* for 15 min at 4 °C. The clarified supernatant was filtered using Whatman No. 1 filter paper to quantify total reducing sugars, cellulase activity, glucoamylase activity and pH [30].

#### 2.4.1. Total Reducing Sugar Test

The total reducing sugar concentration was measured using the DNS method proposed by Miller in 1959 [34]. This method provides a simple and fast way to handle several samples and was used for the optimization process. The determination is centered on the color reaction between reducing sugars and 3,5-dinitrosalicylic acid. The reaction yield was measured as absorbance of the sample at 540 nm using an LLG–uniSPEC 2 spectrophotometer. The total reducing sugar throughout the studies was expressed as grams of sugar per kilogram of fermented substrate (g/kg_fs_).

#### 2.4.2. Enzymes Activity

Cellulase activity was measured using the protocol described by Ghose (1994) and recommended by IUPAC, using filter paper Whatman No. 1 as a substrate [35]. For this determination, a Whatman No. 1 filter paper strip (1 × 6 cm) with a weight of ≈50 mg was used as a substrate. The paper strip was then rolled, placed in a glass tube, and 1 mL of 0.05 M sodium citrate buffer, pH 4.8, was added, immersing and covering the paper, following, 0.5 mL of enzyme solution was added to the same tube. After this step, the tube was vortexed and incubated in a water bath at 50 °C for 60 min. At the end of the incubation, 3 mL of DNS reagent were added, the tubes were also vortexed and incubated in a water bath for 10 min at 95 °C. All the tubes were then placed in a cold water bath for 5 min, and 9 mL of deionized water was added to each tube. Finally, the tubes were vortexed, and the absorbance was measured at 540 nm against reagent blank using an LLG–uniSPEC 2 spectrophotometer. The cellulase activity was estimated, which would have released exactly 2.0 mg of glucose utilizing a plot of glucose liberated against cellulase concentration, was carried out. One FPU/g_ds_ represents the enzyme unit per gram of initial dry solid substrate [24].

Glucoamylase activity was measured using the protocol described by Melikoglu and et al. in 2013 [30]. The glucoamylase activity of the enzyme solutions was estimated by measuring the amount of glucose released per minute using 1 mL potato starch (6% *w*/*v*) as substrate. Glucoamylase activity gave the difference between time zero and 10 min. Throughout the studies, the glucoamylase activity was expressed as U/g_db_ dry basis. One unit (U) was estimated as the enzyme amount required to produce 1 mg of glucose/minute under the assay conditions.

### 2.5. pH Measurements

The pH measurement from SsF experiments was determined with a digital pH-meter (WTW, Weilheim, Germany) at room temperature using the enzymatic extract obtained.

### 2.6. Analytical Assays

During the hydrolyses and fermentations, quantification of glucose, fructose, disaccharide, xylose, arabinose, LA and acetic acids were measured via HPLC (Ultimate 3000 from the company DIONEX (Sunnyvale, CA, USA)). Equipment with the following parameters: a column Eurokat H (300 × 8 mm × 10 µm), company KNAUER; the mobile phase was 0.01 N sulfuric acid with a 0.8 mL/minute rate and a pressure of 65 bar connected with a detector RI–101 (SHODEX, Tokyo, Japan); the injection volume was 10 µL, and the auto-sampler was WPS–3000TSL analytical [33]. All the experiments, hydrolysis and fermentation were made in duplicate and triplicate, and the result are exposed as average ± standard deviation.

## 3. Results and Discussion

### 3.1. Solid State Fermentation, Substrate Optimization and Enzymes Production

In general, the initial culture and the fermentation substrate are well known to influence the production of enzymes. Therefore, in our experiments, WB and WB supplemented with glucose (WB+G) (moisture content was adjusted with glucose solution at a concentration of 30 g/L) were evaluated as substrates for SsF with *A. awamori* to produce enzymes with potential application in enzymatic hydrolyses. During 96 h of fermentation, WB and WB+G were able to support the fungi growth. The SsF control parameters showed a desirable behavior, maintaining the moisture content and a small reduction of the pH values. The initial pH value went from 6.032 ± 0.195 to 5.585 ± 0.119 and from 6.256 ± 0.106 to 5.103 ± 0.151 for WB and WB+G, respectively. Total reducing sugars and enzymatic activity were also monitored. It is remarkable that, at the end of the fermentation, after 96 h, the reducing sugars had similar values of 4.45 ± 0.54 g/kg_fs_ for the simple WB substrate and 4.855 ± 0.837 g/kg_fs_ for WB+G. In addition, an experiment containing the addition of yeast extract was performed. However, the results were worse than WB+G and the same as WB without nutrient supplementation.

**Figure 2:**
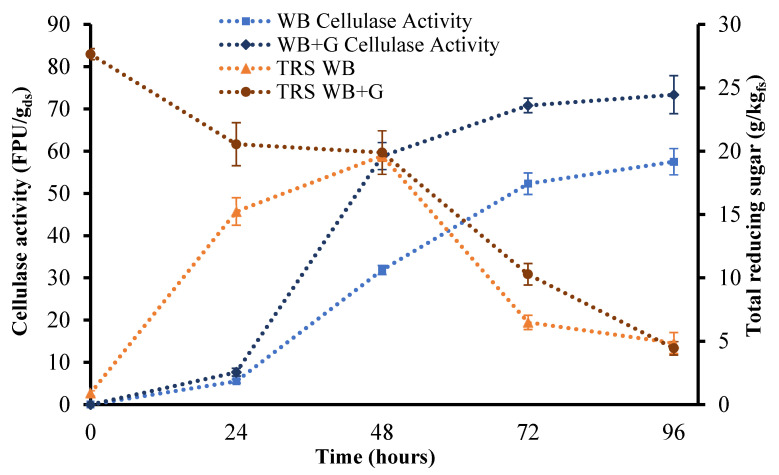
Cellulase activity and total reducing sugar (TRS) for wheat bran fermented by *A. awamori* during 96 h. Square and rhombus represent cellulase activity (FPU/g_ds_); ··■··WB shows wheat bran substrate with sterile deionized water; ··♦··WB+G shows wheat bran substrate with the addition of glucose. Triangle and circle represent total reducing sugar; ··▲··WB shows wheat bran substrate with sterile deionized water; ··●··WB+G shows wheat bran substrate with the addition of glucose.

Figure 2 shows the reducing sugars production and enzyme activities for the SsF of WB and WB+G over 96 h. The fermented WB+G substrate showed the highest cellulase activity, 73.35 ± 4.51 FPU/g_ds_, after 96 h of fermentation. It was followed by fermentation without glucose addition 57.51 ± 3.10 FPU/g_ds_, which showed a high activity after 96 h of fermentation. Additionally, cellulase production depended on the nature of the carbon source and other vital nutrients existing already in the substrate. Other studies evaluating WB as a substrate for SsF found similar cellulase activities using different fungi, with longer fermentation time (up to 120 h). However, these authors supplemented the media with NH_4_SO_4_, KH_2_PO_4_ and yeast extract to increase the nitrogen, carbon or mineral content [24,36,37]. In the literature, filter paper cellulase activities alternated from 1.7 U/g_ds_ [38,39] to 437.5 U/g_ds_ [40]. It is remarkable that, in this paper, we found similar values without supplements, and we set out to produce a fermented material easy to use and adaptable on an industrial scale for hydrolysis with large volumes. In our case, 96 h of fermentation are not enough for maximum cellulase activity, especially for WB. These results indicate that WB can be used by *A. awamori* to grow and produce enzymes without requiring nutrients supplementation.

### 3.2. Effect of Fermentation Time on Enzymes Activity

**Figure 3:**
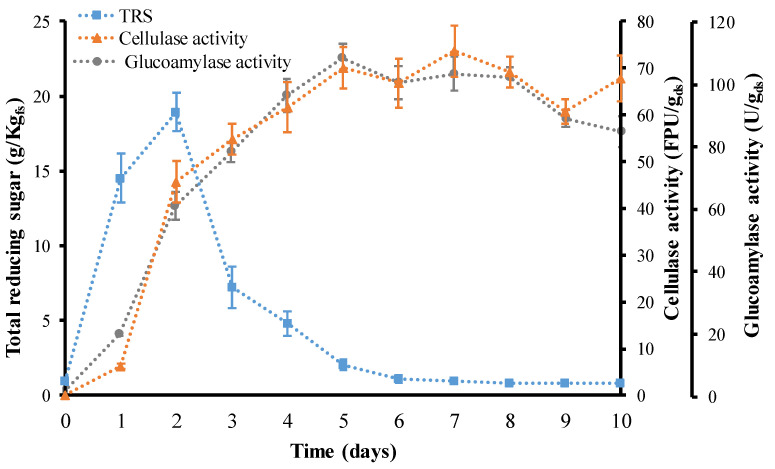
Total reducing sugar (TRS), cellulase and glucoamylase activity during 10 days of fermentation. Square represents total reducing sugars. Triangle represents cellulase activity, and the circle shows glucoamylase activity.

Based on the previously obtained results, WB without nutrient supplementation was selected for the following experiments. During SsF, medium pH, nutrient concentration, temperature, moisture content and physical structure of the raw material continuously change. All these parameters affect microbial growth and are connected directly with enzyme production. Various reports cover different fermentation times for enzyme production via SsF, between 3 and 12 days [24,30,41,42]. Figure 3 shows total reducing sugars, cellulase and glucoamylase activity during 10 days of WB fermentation. In this article, the maximum value for cellulase activity was attained at 7 days of fermentation. In the first 5 days, cellulase activity increased rapidly, reaching 66.96 ± 4.41 FPU/g_ds_, and after that, cellulase activity maintained a slow and constant growth peaking at day 7 with a value of 73.63 ± 5.47 FPU/g_ds_. In addition, cellulase and glucoamylase activities showed a constant and fast evolution until the total reducing sugars were below 2.06 ± 0.35 g/kg_fs_, after 5 days of fermentation. Glucoamylase activity peaked at day 5 with a value of 107.10 ± 2.63 U/g_db_, followed by a constant decrease. These results are in line with those obtained by Kaushik et al. [41], who determined when *Aspergillus lentulus* showed the highest xylanase production via SsF using WB as the substrate and yeast extract as the nitrogen source. The fermentation profile showed a gradual increase in enzyme activity from 24 to 72 h, reaching its maximum value after 96 h and diminishing after 120 h until 144 h of fermentation [41]. Raghuwanshi et al. discussed bioprocessing of enhanced cellulase production using a mutant of *Trichoderma asperellum* RCK2011 via SsF and its application in hydrolysis of cellulose, released cellulases within 48 h of incubation. While the strain exhibited maximum FPase and CMCase after 4 days of fermentation, β–glucosidase production reached the maximum value only after 7 days [43]. A previous study reported that during depletion of the carbon sources in the fermentation medium, the organisms use these hydrolytic enzymes to produce sugars necessary for their own metabolic growth [44]. Various fungi have been reported to produce maximum cellulase at different time intervals; therefore, a direct comparison of time-dependent changes is difficult to perform [45]. These favorable results could be caused by WB, which has provided ample amounts of nutrients in the substrate, such as glucose and starch needed for fungal multiplication and enzyme production.

### 3.3. Hydrolysis Optimization

For the first time in 2008, Melikoglu detailed an integrated new system, using a part of the substrate for the production of hydrolytic enzymes via SsF, and continued with the hydrolysis of the remaining substrate to obtain in final a nutrient-rich hydrolyzate [46]. The nutrient-rich hydrolysate is able to be transformed into a specific product by suitable successive fermentations. Since 2008, many articles have been published analyzing the sequential application of SsF and SmF. It has been well-known that although enzymatic extracts can be used for the hydrolysis, direct addition of fermented solids can make the process more practical and economically feasible [47]. In our case, WBE solids were directly added to OFMSW at different ratios (mass/mass) to evaluate the hydrolysis potential of the WBE. Table 1 illustrates the results obtained during the optimization hydrolysis process. The maximum value of total reducing sugars resulted in a ratio of 4:6 (WBE:OFMSW), 39.53 ± 0.42 g/kg_fs_ after 68 h. An equal ratio of 5:5 obtained a lower result by 12.6% after 68 h than 4:6 ratio. A ratio of 3:7, after 68 h, has a lower result with 3.1% than 4:6 ratio. In addition, a ratio of 2:8 has a lower result at the end of hydrolyzed with 11.7% compared with the best result. In the end, a ratio of 1:9 obtains just 72.1% of total reducing sugar comparing with the best result. These results are quite interesting, indicate that using a 5:5 ratio is not enough new substrate for the enzymes (enzymes remaining available in the substrate). Adding a lower ratio than 4:9, the enzymes were available in the substrate, and hydrolysis of OFMSW occurred but not entirely. However, obtaining a complete hydrolysis and, at the same time, using the enzymes entirely is very difficult to achieve. In our case, using a smaller amount of WBE from an economic point of view is recommended to capitalize on the enzymes obtained from SsF fully. In addition, the optimization hydrolysis results can be influenced by the substrate on which the fungi grew, in our case WB. It is believed that some of the starch could have been located in a lower region of WB where the fungi could not penetrate; also, the growth of the fungi could be uneven throughout the substrate, simply due to the lack of mixing [30].

### 3.4. Hydrolysate Characterization and Lactic Acid Production

**Figure 4:**
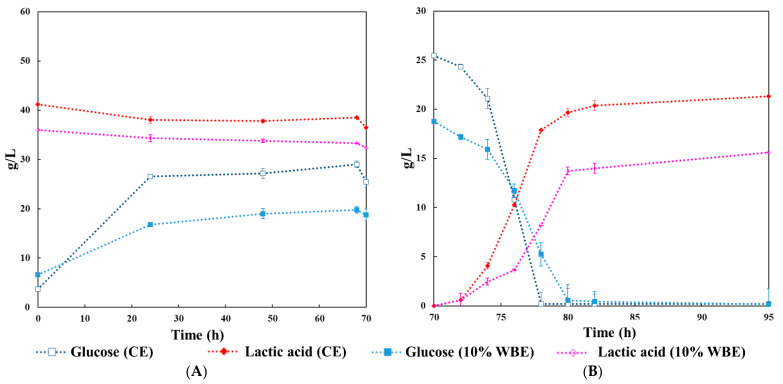
Hydrolysis and subsequent fermentation of OFMSW. (**A**) The graph illustrations the variation in the concentration of glucose and lactic acid during hydrolysis. (**B**) Glucose consumption and lactic acid production during lactic acid fermentation. CE–commercial enzymes; WBE–wheat bran rich in enzyme via SsF.

Table 1 shows the results for the total reducing sugar obtained in hydrolysis optimization, suggesting that OFMSW hydrolyzate could be a suitable substrate for microbial fermentations. Enzymes obtained by SsF were compared with a CE cocktail in two separate hydrolyses. According to the results obtained in the previous section, WBE was added to OFMSW at a ratio of 1:9 (mass/mass). Figure 4 illustrates the profiles for the hydrolysis and successive fermentation of OFMSW, monitoring glucose production/consumption and LA production during the process. Glucose was initially presented in both hydrolyses with concentrations of 6.64 ± 0.19 g/L and 3.68 ± 0.19 for the WBE and the CE, respectively. The higher concentration of glucose for the WBE experiment is the result from the previous SsF of the WB, which liberated sugars. Thus, an addition of 10% WBE increased glucose in the initial hydrolysis substrate. For all hydrolyses, glucose increases considerably during the first 24 h, followed by a slower increase until 68 h with a final value of 19.77 ± 1.56 g/L in the experiment with WBE and 29.00 ± 0.65 g/L in the experiment with CE.

LA and acetic acids were present in both hydrolysates performed. The presence of these acids was detected from the start of the hydrolysis, showing that they were produced by naturally occurring organisms present in the OFMSW before sterilization [3,48]. Racemic LA was initially present in the hydrolysates with concentrations of 35.98 ± 0.98 g/L and 41.22 ± 1.39 g/L in the trials with WBE and CE, respectively. Probst et al. (2015) showed a natural production of an isomeric racemic mixture of LA in kitchen food waste and OFMSW [49]. Although a racemic mixture of LA can produce organic solvents, for example, ethyl lactate, high optical purity is essential for other applications [31,49]. Notably, for polylactic acid production, the ratio D– to L–lactic acid has a significant effect on the final product properties such as its thermal stability, crystallinity, biodegradation and commercially, a combination with a higher quantity of L–lactic acid is used and preferred [50,51].

During LA fermentation in 12 h, glucose was consumed almost entirely by *B. coagulans*. After 25 h of LA fermentation, LA increased by 15.59 g/L in the WBE batch and 21.32 g/L in the CE batch (Figure 4 B). This result indicates that only 73.12% LA was obtained in the WBE batch compared to the CE batch.

Table 2 shows variation in the OFMSW hydrolysate and LA fermentation composition using WBE, and Table 3 shows using CE. Glucose and xylose are the most abundant monosaccharides in lignocellulosic biomass, taking up 60–70 and 30–40% of their hydrolysates, respectively. By reason of the nature of the substrate, differences of this type between batches are difficult to avoid. The *B. coagulans* was able to consume xylose and glucose at the same time. However, in contrast with the case of glucose, the decrease of xylose ceased without being completely depleted [52]. Similar amounts of xylose were diminished in both batches, WBE batch until 7.29 ± 0.32 g/L, and CE batch was diminished until 7.46 ± 0.78 g/L. Contrasting lignocellulosic substrates, such as hardwood and softwoods, with a higher hemicellulose content, the xylose content of OFMSW hydrolysates was lower than the content of glucose [3]. Oppositely, at the start of the hydrolysis, xylose content was higher compared with glucose. An explanation may be most likely because part of the available glucose was fermented by LA bacteria from organisms present in the OFMSW.

Comparing the results from both tables, adding 10% WBE decreased the initial value for acetic acid, LA, xylose, disaccharide and fructose, indicating that the SsF did not experience contamination by LA bacteria [53]. In addition, disaccharides showed a constant decrease in both, hydrolysis and fermentation, reaching 2.65 ± 0.58 g/L for WBE hydrolysis and 1.68 ± 0.61 g/L at the end of fermentation, 2.63 ± 0.90 g/L for hydrolysis with CE, and 1.87 ± 0.75 g/L at the end of fermentation. However, previous articles reported by López-Gómez et al. show no significant variations in the concentration of disaccharides by the end of the fermentation [3,7]. Moreover, Aulitto et al. in 2017 reported that some strains of *B. coagulans* are struggling with the consumption of disaccharides with μmax values 50% lower than those obtained from glucose. This could explain why the concentration of disaccharides did not show a significant change throughout the SmF [54].

### 3.5. Mass Balance

Considering a ratio of 1:9, WBE to OFMSW, and the current SsF bioreactors available, scaling up the whole process can prove to be difficult. In terms of sugars, in order to obtain 1 kg of TRS, considering the value obtained in the optimization section, 35.09 kg of the mixture WBE and OFMSW (1:9) would be needed, more specifically 3.51 kg WBE and 31.58 kg OFMSW.

The results obtained in the previous section indicate that enzymes produced by SsF, mixed in a 1:9 ratio with OFMSW, have a 68.17% glucose release compared to the experiments in which CE was used. In addition, to obtain 1 kg of glucose, a 50.58 kg mix of WBE and OFSMW (ratio of 1:9) would be necessary. Furthermore, considering the same ratio, WBE:OFMSW, 64.14 kg of the mix would be required to produce 1 kg of LA. The amount of substrate required is reduced when using CE. For example, it would be possible to obtain 1 kg of glucose from 34.48 kg of OFMSW and 1 kg of LA from 46.90 kg of OFMSW. Naturally, this higher efficiency of CE can be offset by their cost.

WB is a good substrate for enzyme production, making it widely used and economically suitable. Its physical and chemical characteristics make WB an especially good substrate for SsF process. Thus, the combination of WB and OFMSW in the process takes advantage of their individual characteristics in order to have a process thar provides good enzymatic levels and at the same time exploits the carbohydrate potential in the OFMSW. Future work should target the optimization enzyme production in SsF with tests of different temperatures, moisture contents or even with different mixtures of nutrients (glucose + yeast extract + minerals, etc.) to enhance the hydrolysis step. Additionally, studies using SsF bioreactors which could allow for larger volumes of WB and mixing would definitely provide useful results for the scale up of the system.

Further work could be carried out to investigate WB and OFMSW as important substrates for the fermentation process targeting other valuable compounds. Specially, fermentation products using thermophilic and extremophilic organisms could benefit from such SsF-SmF configuration. An essential advantage of the whole process is the working temperatures for enzyme production (*A. awamori* DSM No. 63272 using 30 °C), the hydrolysis (enzyme hydrolysis using 50 °C), followed by a LA fermentation (*B. coagulans* A166 using 52 °C). This difference in temperature means that the fungi are not activated in hydrolysis, and LA fermentation could start in the same reactor; without the need to sterilize the materials [47]. A challenge may be the autoclaving of substrates, recommended especially for OFMSW, in order to stop spontaneous fermentation with microorganisms already existing in the substrate, which can significantly depreciate its carbohydrate content. A potential alternative to circumvent this issue would be reducing to the maximum storage times or the addition of growth inhibitors during storage. Additionally, studies could be carried using extremophiles during the SmF which could reduce the chances of indigenous bacteria from the waste to grow.

## 4. Conclusions

In this study, the results obtained indicate that WB can be used by *A. awamori* to grow and produce enzymes without requiring nutrients supplementation. The SsF of WB was evaluated to obtain enzymes such as cellulase and glucoamylase. Cellulase and glucoamylase activities were 73.63 ± 5.47 FPU/g_ds_ and 107.10 ± 2.63 U/g_db_ after 7 days and 5 days of fermentation, respectively. Continuing with the hydrolysis of OFMSW, fermented WB solids containing the crude enzymes and mixed with OFMSW in a 1:9 ratio (WBE:OFMSW), showed a 68.17% glucose release compared to the experiments in which CE were used. During the SmF, a 73.12% of LA was obtained in the WBE batch compared to the CE batch. Further work, targeting the optimization of the SsF step, for a better provision of enzymes for the hydrolysis, would enhance these results. Previous reports have studied the conversion of the OFMSW into LA but only employing CE for the hydrolysis step. Nonetheless, utilizing wastes (i.e., WB) for the production of enzymes, instead of simple sugars, as is the case for CE, fits very well in the circular bioeconomy context. Furthermore, the integration of SsF and SmF for the conversion of OFMSW in a process in which only residues are needed as substrates, zero-waste are generated and that utilizes enzymes produced on-site, should represent a more economically attractive process.

## Figures and Tables

**Table 1:** Optimization hydrolysis process of OFMSW with different concentrations of WBE.

Substrate		Total Reducing Sugar (g/kg_fs_)			
WBE (g)	OFMSW (g)	0 h	24 h	48 h	68 h
10	90	4.06 ± 0.23	20.99 ± 0.49	24.81 ± 0.33	28.50 ± 0.49
20	80	5.76 ± 0.26	24.72 ± 0.41	28.85 ± 0.38	34.92 ± 0.43
30	70	6.28 ± 0.27	26.26 ± 0.35	31.96 ± 0.46	38.32 ± 0.41
40	60	9.44 ± 0.33	26.91 ± 0.46	33.18 ± 0.34	39.53 ± 0.42
50	50	9.32 ± 0.30	27.55 ± 0.45	33.02 ± 0.32	34.49 ± 0.47

The total reducing sugar was expressed as grams of sugar per kilogram of fermented substrate (g/kg_fs_). The experiments were made in triplicate, and the values represent the average and standard deviation of them. WBE–fermented wheat bran rich in enzymes; OFMSW–organic fraction municipal solid waste.

**Table 2:** The average concentration of sugars, lactic and acetic acid obtained by hydrolysis and fermentation from OFMSW using WBE (g/L).

		Glucose	Fructose	Disaccharide	Xylose	Arabinose	Lactic Acid	Acetic Acid
Hydrolysis	Initial	6.64 ± 0.19	9.58 ± 0.91	3.99 ± 0.74	10.18 ± 0.42	N.D.	35.98 ± 0.98	5.45 ± 0.11
	Final	19.77 ± 1.56	9.62 ± 0.36	2.65 ± 0.58	9.87 ± 0.13	0.62 ± 0.12	33.30 ± 0.36	5.38 ± 0.51
LA fermentation	Initial	18.77 ± 1.35	8.85 ± 0.31	1.88 ± 0.45	9.29 ± 0.45	0.54 ± 0.16	32.38 ± 0.85	5.30 ± 0.25
	Final	N.D.	7.06 ± 0.56	1.68 ± 0.61	7.29 ± 0.32	N.D.	47.97 ± 0.37	4.98 ± 0.15

The experiments were made in duplicate, and the values represent the average and standard deviation of them. OFMSW–organic fraction of municipal solid waste; WBE–Wheat bran rich in enzymes; N.D.–Not detected.

**Table 3:** The average concentration of sugars, lactic and acetic acid obtained by hydrolysis and fermentation from OFMSW using CE (g/L).

		Glucose	Fructose	Disaccharide	Xylose	Arabinose	Lactic Acid	Acetic Acid
Hydrolysis	Initial	3.68 ± 0.19	10.08 ± 1.41	4.73 ± 0.30	11.14 ± 0.48	N.D.	41.22 ± 1.39	6.16 ± 0.18
	Final	29.00 ± 0.65	10.05 ± 0.57	2.63 ± 0.90	10.79 ± 0.20	0.51 ± 0.15	38.52 ± 0.60	6.03 ± 0.03
LA fermentation	Initial	25.46 ± 1.17	9.15 ± 0.30	2.26 ± 0.39	9.87 ± 0.45	0.50 ± 0.17	36.44 ± 0.52	5.88 ± 0.09
	Final	N.D.	5.28 ± 0.98	1.87 ± 0.75	7.46 ± 0.78	N.D.	57.76 ± 4.41	5.39 ± 0.28

The experiments were made in duplicate, and the values represent the average and standard deviation of them. OFMSW–organic fraction of municipal solid waste; CE–commercial enzymes; N.D.–Not detected.

## Data Availability

The data presented in this study are available within the article. Other data that support the findings of this study are available upon request from the corresponding authors.
